# No Need for Labels: The Autofluorescence of *Leishmania tarentolae* Mitochondria and the Necessity of Negative Controls

**DOI:** 10.1371/journal.pone.0047641

**Published:** 2012-10-15

**Authors:** Elisabeth Eckers, Marcel Deponte

**Affiliations:** Department of Parasitology, Ruprecht-Karls University, Heidelberg, Germany; University of Melbourne, Australia

## Abstract

**Background:**

Fluorescence microscopy is a powerful tool to study the morphology and function of subcellular compartments or to determine the localization of proteins. The method is also regularly used for the analysis of parasitic protists including kinetoplastida.

**Results:**

Here, we report a significant autofluorescence of *Leishmania tarentolae* mitochondria. The autofluorescence, presumably caused by flavoproteins, was detectable using a variety of cell fixation protocols and had a maximum emission at approximately 538 nm. Stable signals were obtained with xenon lamps as a light source and filter sets that are commonly used for the detection of green fluorescent protein.

**Conclusions:**

On the one hand, we present a methodological approach to examine mitochondrial morphology or to study the colocalization of mitochondrial proteins without additional staining or labeling procedures. On the other hand, under certain experimental conditions, mitochondrial autofluorescence can result in false positive signals, demonstrating the necessity to analyze unlabeled cells as negative controls.

## Introduction

Mitochondria from parasitic protists have gained a lot of interest owing to peculiar properties such as RNA editing [1], citric acid cycle alterations [2], [3], apoptotic markers [4], or mitochondrial protein import machineries [5]. Consequently, it is often necessary to stain mitochondria and/or to confirm the subcellular localization of proteins. Direct fluorescence microscopy, using e.g. green fluorescent protein (GFP) [6], and immunofluorescence microscopy (IM) are common methods of choice for these tasks [7]. IM requires (i) a specific primary antibody against the protein of interest and (ii) a reference signal for colocalization. Depending on whether direct or indirect IM is applied, either the target-specific primary antibody or an immunoglobulin class-specific secondary antibody has to be fluorescently labeled [7]. The reference signal is usually generated either by staining an established marker protein with a differently fluorescently labeled antibody, by GFP-tagging, or by a fluorescent dye that accumulates at defined subcellular structures [7]. For example, so-called MitoTracker dyes are commonly used to stain mitochondria [8]. Here, we (i) report autofluorescent subcellular structures in *Leishmania tarentolae* promastigotes, (ii) identify the mitochondrion as the source of the autofluorescence, (iii) determine the biophysical properties of the fluorophore *in situ*, and (iv) provide methodological recommendations for fluorescence microscopy that might be also relevant for other organisms.

## Methods

### Cell culture and fixation protocols

*L. tarentolae* promastigotes (10 ml) were cultured in T-flasks in supplemented BHI medium according to standard protocols as previously described [5], [9]. A thin layer of mid-log phase parasites was dropped on a microscope slide, dried and fixed for 15 min in one of the following solutions: (i) 100% acetone at −20°C, (ii) 20% (v/v) acetone, 80% (v/v) ethanol at −20°C, or (iii) 4% (w/v) paraformaldehyde (PFA) in phosphate buffered saline (PBS) at room temperature. Alternatively, live cell images were recorded after washing the cells three times in 1 ml PBS (1500 g, 15 min, room temperature). The exposure time for the detection of the green fluorescence was 500 ms for fixed and live cell images.

For MitoTracker staining, 5×10^6^ cells were centrifuged (1500 g, 15 min, room temperature), washed once with 1 ml PBS and resuspended in 1 ml BHI-medium containing 1 µM Mitotracker-Red CM-H_2_XRos. Promastigotes were stained for 20 min on a shaker at 27°C, centrifuged and washed three times with PBS before fixation for 20 min with 4% (w/v) PFA in PBS on a shaker at room temperature. After two more washing steps with PBS, cells were centrifuged on cover slips (1500 g, 15 min), mounted on microscope slides using Mowiol medium and analyzed the next day using a Zeiss Axiovert 200 M and the software Axiovision.

### Laser scanning microscopy

PFA-fixed promastigotes were further analyzed by laser scanning microscopy at variable excitation wavelengths using a Zeiss LSM780 and the software ZEN 2010. Z-stacks were collected at Z increments of 0.41 µm and an excitation wavelength of 458 nm. The same excitation was used to record the emission spectra of whole cells, the cytosol, and the mitochondrion *in situ*.

## Results and Discussion

### Detection of autofluorescent structures in *L. tarentolae*

**Figure 1:**
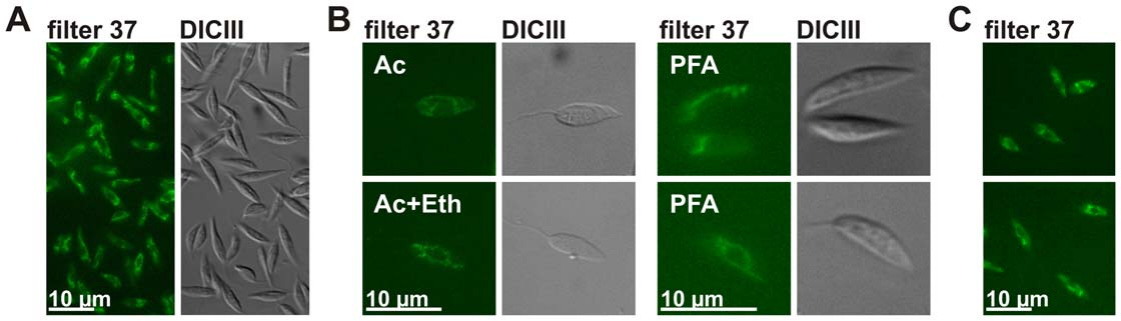
Detection of autofluorescent subcellular structures in *L. tarentolae* promastigotes. Parasites were analyzed by fluorescence microscopy without labeling or staining procedures. (A) Overview of PFA-fixed *L. tarentolae* promastigotes. (B) The following agents were used for alternative fixation protocols: Ac, acetone; Ac+Eth, acetone and ethanol; PFA, PFA in PBS. (C) Live cell images of washed *L. tarentolae* promastigotes without cell fixation. Slides were analyzed with a Zeiss Axiovert 200 M microscope and the software Axiovision. The autofluorescence was detected with the Zeiss filter set 37 (filter 37). Cells were visualized by differential interference contrast (DICIII). A XBO lamp served as a light source.

We recently established the kinetoplastid parasite *L. tarentolae* as a non-opisthokont model organism for the comprehensive analysis of protein import into all four mitochondrial compartments [5]. As a part of this work, we purified four peptide antibodies against different *L. tarentolae* marker proteins. Although these antibodies were well suited for western blot analyses [5], they did not yield satisfactory results in IM studies, particularly because of similar fluorescent structures in unlabeled *L. tarentolae* promastigotes which served as negative controls. Noteworthy, the fluorescence of such distinct subcellular structures in the absence of antibodies (Fig. 1A) was not only seen by eye using a variety of cell fixation protocols (Fig. 1B and Methods), but also without cell fixation (Fig. 1C). Hence, the fluorescence was not caused by external chemicals, but is an intrinsic property of *L. tarentolae*. We subsequently checked different filter sets in order to narrow down the properties of the autofluorescence. Defined structures were detected with the Zeiss filter set 37 (GFP, excitation BP450/50, beam splitter FT480, emission BP510/50) in contrast to filter sets 49 (DAPI, excitation G365, beam splitter FT395, emission BP445/50) and 20 (rhodamine, excitation BP546/12, beam splitter FT560, emission BP575-640). The integrity of all filters was confirmed by a Zeiss employee. In summary, *L. tarentolae* promastigotes possess distinct autofluorescent structures that are detectable with common GFP filter sets.

### Identification of the autofluorescent structures

**Figure 2:**
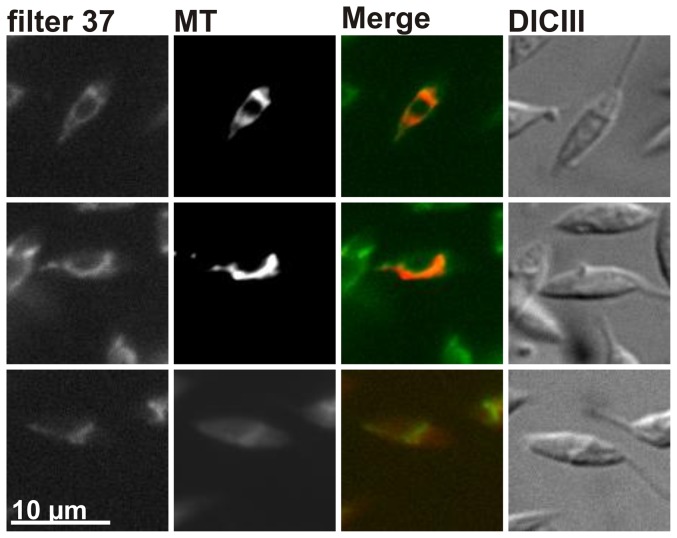
MitoTracker colocalization analyses. *L. tarentolae* promastigotes were stained with a MitoTracker dye before fixation with PFA and analysis by fluorescence microscopy. Slides were analyzed using a Zeiss Axiovert 200 M microscope and the software Axiovision. The autofluorescence was detected with the Zeiss filter set 37 (filter 37). MitoTracker staining was detected with the Zeiss filter set 20 (MT). Cells were visualized by differential interference contrast (DICIII). A XBO lamp served as a light source.

The shape and distribution of the autofluorescent structures was highly similar to the variable morphology of the single *L. tarentolae* mitochondrion: In dividing promastigotes the mitochondrion has a rather symmetric and circular shape, whereas in non-dividing cells the mitochondrion becomes a single asymmetric tubule [10]. In order to confirm an autofluorescence of the *L. tarentolae* mitochondrion, we subsequently performed a colocalization experiment with a MitoTracker dye. A high degree of colocalization was observed between the autofluorescent signal, detected with the GFP filter set 37, and the MitoTracker signal, detected with the rhodamine filter set 20 (Fig. 2). We therefore conclude that the autofluorescent signal derives from the *L. tarentolae* mitochondrion which can be visualized without prior staining. Please note that MitoTracker dyes are rather expensive and have a limited shelf life. Moreover, their uptake usually varies between individual cells (i.e. Fig. 2), whereas the autofluorescence is an intrinsic property of all cells and does not require expensive labels. In summary, mitochondrial autofluorescence provides an excellent reference signal for colocaliation studies in *L. tarentolae* promastigotes.

### Further applications and instrumental settings

**Figure 3:**
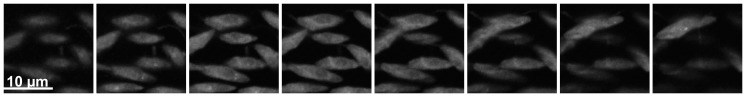
Mitochondrial morphology studied by laser scanning microscopy. PFA-fixed *L. tarentolae* promastigotes (Fig. 1A,B) were analyzed by laser scanning microscopy using a Zeiss LSM780 microscope and the software ZEN 2010. Z-stacks at Z increments of 0.41 µm are shown from left to right. A 458 nm laser served as a light source.

Next, we analyzed whether the autofluorescence can be also used for morphological studies of the single *L. tarentolae* mitochondrion. Furthermore, we compared different instruments and instrumental settings in order to determine the optimum experimental conditions for the detection of the autofluorescence. The morphology was studied on a LSM780 confocal laser scanning microscope using an excitation at 458 nm (Fig. 3). Other excitation wavelengths did not result in defined autofluorescent subcellular structures in accordance with the filter sets described above. Z-stacks of the *L. tarentolae* mitochondrion were in good agreement with previous morphology models based on IM and rhodamine 123 staining [10]. Noteworthy, using the LSM780 microscope, the fluorophore was more labile, and the autofluorescence was lost within a few seconds even at low laser intensities in contrast to the experiments summarized in Figs. 1 and 2. A subsequent comparison of different settings and microscopes revealed that the autofluorescence intensity and photobleaching kinetics highly depended on the light source. Brightest and relatively stable signals were obtained when a XBO (xenon) lamp was used (Fig. 1 and 2). In contrast, HBO (mercury) lamps or laser (Fig. 3) resulted in rather rapid photobleaching. We therefore recommend the use of XBO lamps for future studies on mitochondrial autofluorescence. Under such conditions, the signal-to-noise ratio could be also suited for morphological or metabolic studies without additional labels.

### Characterization of the fluorophore and comparison with mammalian cells

**Figure 4:**
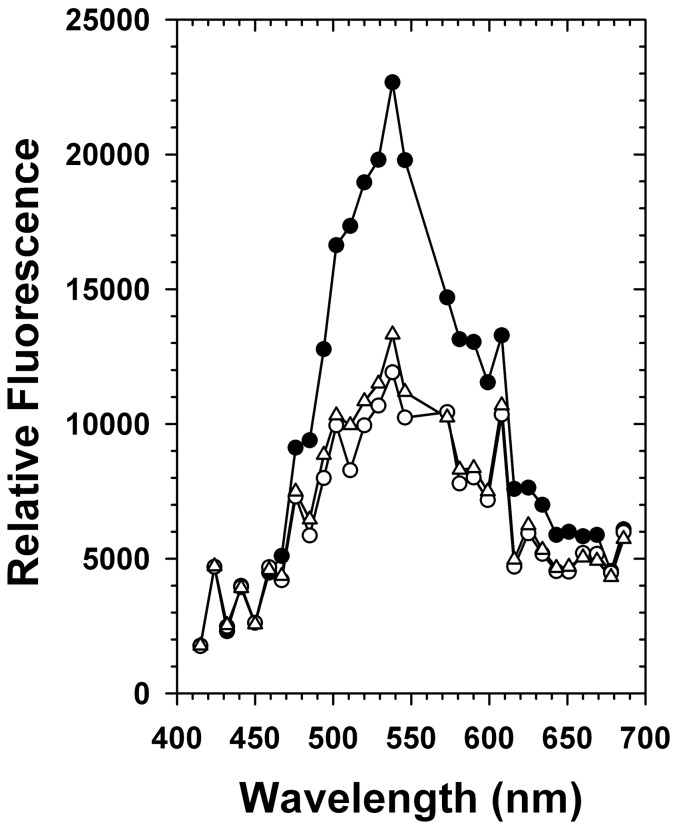
Fluorescence emission spectra. The fluorophore properties of PFA-fixed *L. tarentolae* promastigotes (Fig. 1) were analyzed *in situ* by laser scanning microscopy using a Zeiss LSM780 microscope and the software ZEN 2010. Emission spectra of the cytosol (open circles), mitochondria (closed circles), and whole cells (open triangles) are shown. A 458 nm laser served as a light source.

In order to obtain more information on the properties of the fluorophore, we compared the emission spectra of fixed whole cells, the cytosol, and the mitochondrion *in situ* (using the LSM780, an excitation at 458 nm, and a 458/561 nm beam splitter). The shapes of all spectra were quite similar, revealing two pronounced emission maxima around 538 nm and 608 nm (Fig. 4). The fluorescence of mitochondria at 538 nm was twice as high as for the cytosol. Furthermore, the ratio of the fluorescence intensities at 538 and 608 nm was 1.5 times higher in the mitochondrial fraction than in the cytosol. Please note that these numbers are rather underestimates owing to photobleaching effects caused by the laser of the LSM780 (see above). In summary, a major fluorophore of *L. tarentolae* with an excitation at approx. 458 nm and an emission maximum at approx. 538 nm is distributed throughout the cell but is highly enriched in the mitochondrion.

In contrast to kinetoplastida, mitochondrial autofluorescence has previously been reported for diverse mammalian cell types [11]–[15]. Among the intracellular repertoire of molecules with delocalized π electrons, NADH, heme/cytochromes, and FAD/flavoproteins are likely sources for the spectra in Fig. 4 (since mitochondria are enriched in these fluorophores owing to the respiratory chain as well as numerous catabolic flavoproteins). Accordingly, previous studies on mammalian cells suggested that NAD(P)H and flavoproteins are usually the most important cellular fluorophores in the visible spectrum [11]–[14], [16]–[18]. Their influence can often be distinguished based on their emission spectra: NAD(P)H has a maximum emission around 450 nm, whereas many flavin fluorophores, such as dihydrolipoamide dehydrogenase, have a maximum emission between 500 and 550 nm [14], [16]–[20]. This area of the spectrum correlates well with the observed maximum at 538 nm in Fig. 4. Thus, distinct flavoproteins are candidates for the major autofluorescence of the *L. tarentolae* mitochondrion. Among the flavoproteins, dihydrolipoamide dehydrogenase of the 2-oxo acid dehydrogenase complexes (e.g. fueling the citric acid cycle) and the electron transfer flavoprotein (required for β-oxidation) were estimated to account for approx. 75% of the mitochondrial flavoprotein fluorescence in isolated rat liver mitochondria [17], [18]. The fluorescence of many other flavoproteins was suggested to be either quenched or of little relevance owing to lower physiological concentrations [17], [18]. Dihydrolipoamide dehydrogenase therefore might also be a candidate for the mitochondrial autofluorescence of *L. tarentolae*, even though kinetoplastida do not necessarily require functional 2-oxo acid dehydrogenase complexes [21]. The causative fluorophore of the emission at approx. 608 nm is less obvious, but diverse cytochromes might have maxima around this wavelength. Of note, if the autofluorescence is linked to the mitochondrial electron transport chain, the detection method described here might be useful for the analysis of (potential) respiratory chain inhibitors.

## Conclusions

We identified mitochondrial autofluorescence as an intrinsic property of *L. tarentolae* promastigotes and demonstrated its suitability for general applications in fluorescence microscopy. In addition, we determined the optimum instrumental settings and characterized the fluorophore properties. A significant mitochondrial autofluorescence has, to our knowledge, never been mentioned in previous studies on kinetoplastid parasites. This might be due to the rapid photobleaching caused by the commonly used HBO lamps. However, when analyzing the general literature on fluorescence microscopy with kinetoplastid parasites, we realized that negative controls of unlabeled cells were usually neither shown nor mentioned. Thus, false positive signals cannot be fully excluded, especially for low signal-to-noise ratios. We would therefore like to suggest the inclusion of negative controls for the standard presentation of fluorescently labeled kinetoplastid parasites, in particular, when microscopes with XBO lamps are used and/or mitochondrial structures are studied. Moreover, it is quite likely that a mitochondrial autofluorescence is not only restricted to the reported organisms, but can be found in most eukaryotes. Thus, the presented data might also have more general implications for fluorescence studies in eukaryotes.

## References

[1] Knoop V (2011) When you can't trust the DNA: RNA editing changes transcript sequences. Cell Mol Life Sci 68: 567–586. 20938709 10.1007/s00018-010-0538-9 PMC11114842

[2] Olszewski KL, Mather MW, Morrisey JM, Garcia BA, Vaidya AB, et al (2010) Branched tricarboxylic acid metabolism in Plasmodium falciparum. Nature 466: 774–778. 20686576 10.1038/nature09301 PMC2917841

[3] Rivière L, Moreau P, Allmann S, Hahn M, Biran M, et al (2009) Acetate produced in the mitochondrion is the essential precursor for lipid biosynthesis in procyclic trypanosomes. Proc Natl Acad Sci USA 106: 12694–12699. 19625628 10.1073/pnas.0903355106 PMC2722340

[4] Deponte M (2008) Programmed cell death in protists. Biochim Biophys Acta 1783: 1396–1405. 18291111 10.1016/j.bbamcr.2008.01.018

[5] Eckers M, Cyrklaff M, Simpson L, Deponte M (2012) Mitochondrial protein import pathways are functionally conserved among eukaryotes despite compositional diversity of the import machineries. Biol Chem 393: 513–524. 22628314 10.1515/hsz-2011-0255

[6] Deponte M (2012) GFP tagging sheds light on protein translocation: implications for key methods in cell biology. Cell Mol Life Sci 69: 1025–1033. 22349212 10.1007/s00018-012-0932-6 PMC11115126

[7] Griffiths G (1993) Fine structure immunocytochemistry. Heidelberg: Springer-Verlag.

[8] Poot M, Zhang YZ, Krämer JA, Wells KS, Jones LJ, et al (1996) Analysis of mitochondrial morphology and function with novel fixable fluorescent stains. J Histochem Cytochem 44: 1363–1372. 8985128 10.1177/44.12.8985128

[9] Simpson L, Frech GC, Maslov DA (1996) RNA editing in trypanosomatid mitochondria. Methods Enzymol 264: 99–121. 8965731 10.1016/s0076-6879(96)64012-9

[10] Simpson L, Kretzer F (1997) The mitochondrion in dividing Leishmania tarentolae cells is symmetric and circular and becomes a single asymmetric tubule in non-dividing cells due to division of the kinetoplast portion. Mol Biochem Parasitol 87: 71–78. 9233674 10.1016/s0166-6851(97)00044-3

[11] Benson RC, Meyer RA, Zaruba ME, McKhann GM (1979) Cellular autofluorescence-is it due to flavins? J Histochem Cytochem 27: 44–48. 438504 10.1177/27.1.438504

[12] Aubin JE (1979) Autofluorescence of viable cultured mammalian cells. J Histochem Cytochem 27: 36–43. 220325 10.1177/27.1.220325

[13] Andersson H, Baechi T, Hoechl M, Richter C (1998) Autofluorescence of living cells. J Microsc 191: 1–7. 9723186 10.1046/j.1365-2818.1998.00347.x

[14] Chorvat D Jr, Bassien-Capsa V, Cagalinec C, Kirchnerova J, Mateasik A, et al (2004) Mitochondrial autofluorescence induced by visible light in single rat cardiac myocytes studied by spectrally resolved confocal microscopy. Laser Physics 14: 220–230.

[15] Davis RW, Timlin JA, Kaiser JN, Sinclair MB, Jones HD, et al (2010) Accurate detection of low levels of fluorescence emission in autofluorescent background: Francisella-infected macrophage cells. Microsc Microanal 16: 478–487. 20569528 10.1017/S1431927610000322 PMC2944771

[16] Duchen MR, Surin A, Jacobson J (2003) Imaging mitochondrial function in intact cells. Methods Enzymol 361: 353–389. 12624920 10.1016/s0076-6879(03)61019-0

[17] Kunz WS, Kunz W (1985) Contribution of different enzymes to flavoprotein fluorescence of isolated rat liver mitochondria. Biochim Biophys Acta 841: 237–246. 4027266 10.1016/0304-4165(85)90064-9

[18] Kunz WS (1986) Spectral properties of fluorescent flavoproteins of isolated rat liver mitochondria. FEBS J 195: 92–96. 10.1016/0014-5793(86)80137-5 3753688

[19] Romashko DN, Marban E, O'Rourke B (1998) Subcellular metabolic transients and mitochondrial redox waves in heart cells. Proc Natl Acad Sci USA 95: 1618–1623. 9465065 10.1073/pnas.95.4.1618 PMC19119

[20] Huang S, Heikal AA, Webb WW (2002) Two-photon fluorescence spectroscopy and microscopy of NAD(P)H and flavoprotein. Biophys J 82: 2811–2825. 11964266 10.1016/S0006-3495(02)75621-X PMC1302068

[21] van Weelden SW, van Hellemond JJ, Opperdoes FR, Tielens AG (2005) New functions for parts of the Krebs cycle in procyclic Trypanosoma brucei, a cycle not operating as a cycle. J Biol Chem 280: 12451–12460. 15647263 10.1074/jbc.M412447200

